# Psychological well-being and illness perceptions in patients with hypopituitarism

**DOI:** 10.1007/s11102-021-01131-w

**Published:** 2021-02-19

**Authors:** Tessa N. A. Slagboom, Jan Berend Deijen, Christa C. Van Bunderen, Hans A. Knoop, Madeleine L. Drent

**Affiliations:** 1 Section of Endocrinology, Department of Internal Medicine, Amsterdam UMC, Vrije Universiteit Amsterdam, Amsterdam Neuroscience, De Boelelaan 1117, 1081 HV Amsterdam, The Netherlands; 2 Section of Clinical Neuropsychology, Department of Clinical, Neuro- & Developmental Psychology, Faculty of Behavioural and Movement Sciences, Vrije Universiteit, Van der Boechorstraat 7, 1081 BT Amsterdam, The Netherlands; 3 Hersencentrum Mental Health Institute Amsterdam, Amsterdam, The Netherlands; 4 Division of Endocrinology, Department of Internal Medicine, Radboud University Medical Center, Nijmegen, the Netherlands; 5 Department of Medical Psychology, Amsterdam UMC, University of Amsterdam, Meibergdreef 9, 1105 AZ Amsterdam, The Netherlands

**Keywords:** Hypopituitarism, Pituitary, Well-being, Mood, Mood state, Illness perceptions, Psychology, Psychological complaints, Depression, Causal attribution

## Abstract

**Objective:**

The primary aim of the current study was to objectify a spectrum of persisting subjective psychological complaints in patients with hypopituitarism, at least six months after normalizing of the hormonal disturbances. Also, gender differences on these outcomes were investigated. The secondary aim was to identify illness perceptions and causal attributions within this patient group.

**Methods:**

A total of 42 adult participants (60% females) with treated hypopituitarism once filled out a number of psychological questionnaires. The Profile of Mood States (POMS) and the Hospital Anxiety and Depression Scale (HADS) assessed mood and the Symptom Checklist-90 (SCL-90) and the Work and Social Adjustment Scale (WSAS) assessed well-being. Illness perceptions were identified using the Illness Perceptions Questionnaire-Brief Dutch Language Version (IPQ-B DLV) and causal attributions by using the Causal Attribution List (CAL). Patient outcomes were compared to reference values of healthy norm groups.

**Results:**

Participants scored significantly worse on the POMS depression, anger, fatigue and tension subscales, the SCL-90 psychoneuroticism, depression, inadequacy of thinking and acting and sleeping problems subscales and all subscales of the WSAS when compared to reference data. Women also scored worse on depression (HADS) and somatic symptoms (SCL-90). Compared to other illnesses, patients with hypopituitarism have more negative and realistic illness perceptions on consequences, timeline, identity and emotions. Participants attributed their complaints more to physical causes than psychological causes.

**Conclusion:**

Despite normalization of hormonal disturbances, patients with hypopituitarism in general can still experience problems during daily living, such as negative mood states and a decreased psychological well-being.

## Introduction

Receptors of hormones of the pituitary axes are present in the central nervous system [1–6] and can thereby have an effect on the structure and function of the brain. For example, the GH/IGF-1 axis is known to play an important role in neuronal development and function [7]. Several studies found that deficiencies in the pituitary axes have a negative effect on central functions such as cognition and mood [8–16].

Hormonal disturbances in hypopituitarism are treated with hormonal replacement therapy. Although replacement therapy has a positive effect on some of the clinical manifestations and comorbidities related to hypopituitarism [17–23] treated patients still regularly experience problems during daily living. These problems are similar to the known symptoms related to hypopituitarism and include fatigue, pain, physical sexual dysfunction, impaired cognitive functioning and sleeping problems [9, 24–29]. A study of Biermasz et al. [30] found altered sleep characteristics in patients treated for non-functioning pituitary adenoma (NFPA), including disturbed distribution of sleep stages and disturbed circadian movement rhythm. These disturbed sleep characteristics led to increasing fatigue and could be contributing to a decreased quality of life (QoL) and cognitive impairment. In a review, Romijn [31] stated that sleep disorders can arise after compression of the optic chiasm, postoperative radiotherapy and/or hypothalamic dysfunction. Insufficient sleep leads to a decline in cognition by slowing of response speed and attention in general, but might also affect specific cognitive systems [32].

Furthermore, long-term cured patients with pituitary pathology still experience a lasting decrease in psychological well-being, which is confirmed by studies showing increased psychopathology and maladaptive personality traits [33, 34]. For example, these patients show more anxiety, apathy and irritability. Previous studies found negative effects on mood in patients cured of different pituitary diseases such as acromegaly, Cushing’s disease, NFPA and microprolactinoma [29, 33, 35–37]. In one of these studies, hypopituitarism appeared to be an independent predictor for mood problems such as anxiety, depression and mental fatigue [29] but in other studies, the presence of hypopituitarism did not affect mood [33, 35, 36]. Moreover, impaired QoL is found in patients with long-term treatment of pituitary pathology [29, 35–37]. In one of their studies, Andela et al. [24] organized four focus groups of patients with different pituitary diseases which discussed QoL aspects. Outcomes were categorized according to the biopsychosocial approach of QoL. Physical complaints included fatigue, pain, sleeping disorders and cognitive problems. Psychological problems were less distinct and although all pituitary patient groups experienced stress, anxiety and depressive symptoms were only found in some groups. The mentioned study also reported social problems: the majority had work related problems, limitations in leisure activities and experienced a negative influence on partner relationship. A study of Van Aken et al. [29] showed that in long-term treated Cushing’s disease (CD) the presence of hypopituitarism was an independent predictor of a decrease in QoL, especially concerning mental problems such as anxiety, depression, reduced motivation and mental fatigue. A deterioration in psychological well-being was also found in a study of treated non-functioning pituitary adenoma (NFPA), in which hypopituitarism appeared to be an independent predictor for impaired sleep, poor social functioning and role limitations due to physical problems [36]. The decrease in psychological well-being found in patients treated for pituitary pathology could be due to treatment imperfections and/or structural brain abnormalities due to hormonal under- and/or overproduction [38–40]. Another factor that may affect psychological well-being are illness perceptions.

Illness perceptions refer to the personal ideas and beliefs that patients have about their illness and affect the way they interpret and respond to it. According to the Common-Sense-Model (CSM) of self-regulation [41, 42], illness perceptions are processed in parallel through three stages: representation, coping and appraisal. First, situational stimuli will lead to both cognitive and emotional representations of the illness (“illness perceptions”). These representations then guide coping procedures and will lead to certain behaviours. Finally, an appraisal is made about the efficacy of the coping efforts. Since the outcome of the third stage (the appraisal) is then again used as input for the (change in) formation of illness perceptions and coping strategies, a continuous feedback loop arises.

One aspect of the patients’ illness representations is formed by causal attributions: a patients’ opinion concerning the cause of their illness. Causal attributions thereby reflect the belief about their personal control over the illness and are a known determinant of successful coping or helplessness, depression and disability [43, 44].

Given that all components rely on patients’ own subjective beliefs and expectations, illness representations can deviate from medical facts. Previous studies indicate that negative illness perceptions are associated with a decrease in QoL after long-term treatment of pituitary pathology [45, 46]. By identifying these illness perceptions, discrepancies between the beliefs of patients and medical facts about the illness can be clarified, which may improve perceptions, self-management and illness outcomes [47–49]. This in turn may lead to an improved QoL [50, 51].

Altogether, patients treated for pituitary pathology may still experience problems during daily living, which may lead to a decreased psychological well-being. While previous studies concerning this field focused on specific aetiologies of hypopituitarism, such as acromegaly and Cushing’s disease, this study included a heterogeneous group of adult patients with hypopituitarism. These insights are of importance because they can provide a better understanding about the course and expected long-term complaints during daily living that patients treated for hypopituitarism might encounter in general. This heterogeneous group represents clinical practice, during which endocrinologists will face patients with all kinds of aetiologies, degree of illness and treatments of hypopituitarism.

Therefore, the primary aim of current study was to objectify a spectrum of persisting subjective psychological complaints in adult patients with hypopituitarism, at least six months after normalizing of the hormonal disturbances. It was hypothesized that these patients show subnormal scores on mood and psychological well-being tests when compared to healthy reference groups. Since previous studies [27, 52] have shown a difference in outcome measures for gender in treated hypopituitarism patients, comparisons with gender-related reference groups were made.

The secondary aim was to identify illness perceptions and causal attributions within this patient group. In the future, these identified psychological complaints together with illness perceptions and causal attributions could form a basis for a better understanding of the long-term problems of hypopituitarism and treatment improvements.

## Material and methods

### Participants

The patient group is already described in a previous article of Slagboom et al. [27]. All 42 participants were outpatients of the section of Endocrinology, department of Internal Medicine, Amsterdam UMC, Vrije Universiteit Amsterdam. Participants needed to meet the following criteria: hypopituitarism (a deficit of ≥ 1 of the pituitary hormones), at least six months after stabilizing hormone levels, and age between 18–70 years. Exclusion criteria were: mentally retarded, suffering from dementia or severe (cognitive) illness or chronic use of medication that affects consciousness. Participants had an outpatient clinic appointment at least two times a year and during this appointment pituitary function was assessed and hormonal deficits were suppleted according to international guidelines. Laboratory results of hormone levels of participants during last check-up showed that mean free thyroxine (fT4) was 17.66 pmol/L (*SD* = 2.93, reference range = 12 – 22 pmol/L), mean standardized insulin-like growth factor-1 (IGF-1) was 0.19 (*SD* = 1.16, reference range = -2.00 – 2.00) and mean testosterone was 19.38 nmol/L (SD = 9.00, reference range =  > 8.00 nmol/L). The group of participants consisted of 17 (40%) males and 25 females, with ages ranging from 21 to 70 years, and a mean age of 49 years (*SD* = 15). Duration of disease ranged from 5 to 63 years, with a mean of 22 years (*SD* = 12). The number of pituitary hormone deficiencies ranged from 1 to 5, with a median of 3.5 and 90% of the participants had panhypopituitarism, e.g. ≥ 2 pituitary hormone deficiencies. Number of pituitary axis deficiencies were as followed: adrenocorticotrophin hormone (ACHT)-cortisol: 32 (76%), thyroid-stimulating hormone (TSH)-thyroxin: 34 (81%), follicle stimulating hormone/luteinizing hormone (FSH/LH)-estradiol/testosterone: 30 (71%) deficiency, growth hormone (GH)- insulin-like growth factor (IGF-1): 32 (76%) and antidiuretic hormone (ADH): 14 (33%). For ACTH-cortisol, TSH-thyroxin and ADH deficiency, all patients received hormone substitution. For FSH/LH-estradiol/testosterone deficiency, 19 of 30 participants received hormone substitution because only premenopausal women were substituted. Four participants with GH-IGF-1 deficiency did not receive GH substitution: two had a contraindication due to history of malignancy, one due to a polyp and one did not receive it by own choice. Eighty-two percent of the participants had an acquired form of hypopituitarism and most reported diagnoses included non-functioning pituitary adenoma (24%), craniopharyngioma (14%), acromegaly (12%), Cushing’s disease (12%) and prolactinoma (10%). Eighty-three percent of the participants previously received surgery, 36% received cranial radiotherapy and 5% received chemotherapy. Comorbidity appeared in 26 (62%) participants and included hypertension (45%), type 2 diabetes mellitus (10%), epilepsy (10%), dyslipidaemia (7%), osteoporosis (7%) and asthma (5%). Seventy-one percent of the participants used other medications than suppleted pituitary hormones and most frequently reported medications were antihypertensive (33%), statin (21%), antiepileptic (12%), diabetic (10%) and antidepressant (2%). Participants did not receive a compensation for participating in the study. The protocol was approved by the Medical Ethical Committee of the Amsterdam University Medical Center, Vrije Universiteit Amsterdam.

### Materials

All questionnaires were computerized and filled in with the Philips VitalHealth Questmanager Software®.

#### Subjective well-being

Prior to completing questionnaires, a short interview was held. During the interview, the investigator asked an open question: “Do you experience any problems during daily living, compared to the period before the hypopituitarism?”. The spontaneously named problems were written down and after all data was collected, one of the investigators made categories based upon most common named complaints. These complaints formed a measure of subjective well-being.

#### Mood

Mood was assessed by two questionnaires: the shortened Dutch version of the Profile of Mood States (POMS) and the Hospital Anxiety and Depression Scale (HADS). During completing the POMS [53], the participant are shown 32 moods states and are asked to indicate how often in the last two weeks they felt like this. Rating is on a 5-point scale, ranging from 0 (not at all) to 4 (very). Outcome measures are depression, anger, fatigue, vigor and tension with a respectively maximum score of 48, 42, 36, 30 and 36. Higher scores indicate a worse mood state for depression, anger, fatigue and tension and a positive mood state for vigor. One study regarding the shortened Dutch version of the POMS found the Cronbach’s **α** reliability coefficient to be between 0.82 and 0.91 for the five mood states. The present data were compared with results of this study supplying reference data of 481 healthy Dutch men and 491 healthy Dutch women registered in a general practice [54]. To measure clinical depression and anxiety instead of mood states, the HADS [55] was used. The HADS has been validated in 3491 females and 2698 males between 25 and 65 years registered with three general practices in North West England [56]. The questionnaire consists of 14 items and the participants are asked to indicate to which extent they feel like the item in the past week on a 4-point scale, ranging from 0 to 3. Outcome measures are depression and anxiety, and both have a maximum score of 21 (max total score of 42) with higher scores representing more anxiety and/or depression. Comparisons were made with above mentioned reference values of a sex-related general British population [56].

#### Well-being

Two questionnaires were used to measure well-being: Symptom Checklist (SCL-90) and the Work and Social Adjustment Scale (Sociale Aanpassingslijst; SAL). The SCL-90 [57] is a widely used multidimensional scale used to measure different physical and psychological complaints experienced in the last week. The 90 items are rated on a 5-point Likert scale, varying from 0 (not at all) to 4 (extremely). Outcome measures are total score (psychoneuroticism, 90–450) and 8 subscales with varying scores: agoraphobia (7–35), anxiety (10–50), depression (16–80), somatic symptoms (12–60), inadequacy of thinking and acting (9–45), interpersonal sensitivity (18–90), hostility (6–30) and sleeping problems (3–15). The Dutch version of the SCL-90 has proven to have good internal consistency (**α** > 0.80), sensitivity as well as discriminating and predictive validity, while scores are hardly influenced by background variables such as gender, age, educational level or social desirability. Reference values are derived from the manual of the SCL-90 and include a healthy Dutch population of 2394 (50% male) participants aged between 20 and 65 years [58]. The SAL is a Dutch version of the Work and Social Adjustment Scale (WSAS) [59, 60] and was used to measure the influence of the illness on work and daily living. The WSAS has been reported to be a valid and reliable measure of social and occupational functioning in both mental and physical disorders, with good test–retest reliability and internal validity (Cronbach’s **α** 0.96) [60]. The questionnaire contains 5 items about the impairment in functioning due to the illness, noticed during daily living. Items ranging from 0 (no impairment) to 8 (very severe impairment), with a maximum score of 40 and higher scores indicate more influence of the illness on work and daily living. Outcome measures are total score, work, home management, social leisure, private leisure and close relationships. Reference values are derived from a healthy population consisting of 83 participants from a British local community [61].

##### Illness-perceptions

Illness perceptions were measured using The Brief Illness Perception Questionnaire Dutch Language Version (IPQ-B DLV). The IPQ-B DLV [62] is a shortened version of the IPQ [64] and measured illness perceptions on 8 different closed items and 1 open question. Closed questions are rated on a 10-point Likert scale, ranging from 0 (not at all) to 10 (extremely). Outcome measures are: five cognitive representations: identity (label of the disease and associated symptoms), consequences (beliefs about illness effects and outcomes), timeline (expected duration of illness), personal control (over illness), treatment control (beliefs about treatment effect on illness), two emotional representations: concern (about the illness) and emotions (extent to which the illness affects mood) and comprehensibility (extent to which patients believe to understand their illness). Since the IPQ has been reported to have high test–retest reliability and good concurrent and predictive validity, it is considered a valid and reliable measure of illness perceptions in multiple diseases [63]. The present scores on the closed items are compared to groups with other illnesses described by Broadbent et al. [63] consisting of 309 asthma patients, 119 diabetes patients, 103 patients with myocardial infarction and 49 participants with a cold. After the 8 closed items, an open question is asked: “Please state the 3 most important factors which, in your opinion, caused your disease, in order of importance”. Outcome measure from the open question is causal and is divided into different categories: pituitary pathology, treatment shortcoming, stress, psychological, other disease or other attribution.

#### Causal attributions

The Causal Attributions List (CAL) measures the degree in which patients attribute their complaints to a physical or psychological cause and consists of 10 items on a 4-point scale, ranging from 1 to 4. The same items were used as in a study of Vercoulen et al. [64] about chronic fatigue. Additionally, two extra physical attribution items were added: one concerning hormonal disturbances and one item about the persistence of systems due to a physical cause. Outcome measures are physical and psychological attributions, and scores range from 5 (none) to 20 (strong). Cronbach’s **α** reliability coefficient has been reported to be 0.71 and 0.75 for physical respectively psychological attributions [65]. Present outcomes are compared to a group of 50 patients with multiple sclerosis and a group of 51 patients with chronic fatigue syndrome from a study of Vercoulen et al. [64].

### Procedure

The procedure is already described in a previous article of Slagboom et al. [27]. Consecutive outpatients of the section of Endocrinology, department of internal medicine of the Amsterdam UMC, Vrije Universiteit Amsterdam received an invitation letter from their treating physician to participate in the study. In the weeks hereafter patients were called and an appointment was made. Participants filled in a psychological test battery once in a quiet room, mostly combined with a visit at the outpatient clinic. All questionnaires were filled out on a research computer. Testing time was estimated at 45 min and an investigator (student) was present at all times.

### Data analysis

Statistical analyses were performed with IBM SPSS® Statistics version 22.0. Basic features of the data were provided by descriptive statistics and expressed as the median or *M* ± *SD*. Primary study parameters of psychological well-being were compared with reference data by using Chi-squared tests *χ*^2^ (categorical data), one sample *t*-tests or one sample Wilcoxon signed rank test *Z* (unpaired numerical data). For the HADS, the median instead of the mean was given and used for nonparametric testing. Concerning the CAL, comparisons with reference data (dichotomous reference data were derived from sample size and percentages) were made by *χ*^2^ tests. Since sample sizes are below *n* = 20 for the male group, effect sizes were calculated as corrected effect size *Hedges’ g*: (M_1_-M_2_)/SDpooled and interpreted following Cohen [65]; small (*g* = 0.2), medium (*g* = 0.5) or large (*g* = 0.8). For the HADS, effect sizes *r* were calculated as *Z/*√(*N*) and interpreted following Bartz [66]; very low (*r* < 0.20), low (*r* = 0.20 to 0.40), moderate (*r* = 0.40 – 0.60), strong (*r* = 0.60 – 0.80) or very high (*r* > 0.80). To determine the relationship between different aspects of illness perceptions and psychological well-being, Spearman’s correlations (***ρ***) were computed.

## Results

Of the 101 invited participants, 42 (42%) participated. There was no missing data. Differences on patient characteristics and demographics between participants and non-participants are previously described by Slagboom et al. [27]. The only found differences were that compared to non-participants, participants more often had an acquired form of hypopituitarism (*χ*^*2*^(1) = 6.37, *p* = 0.01) and a history of surgery (*χ*^*2*^(1) = 15.82, *p* < 0.01).

### Subjective well-being

**Fig. 1:**
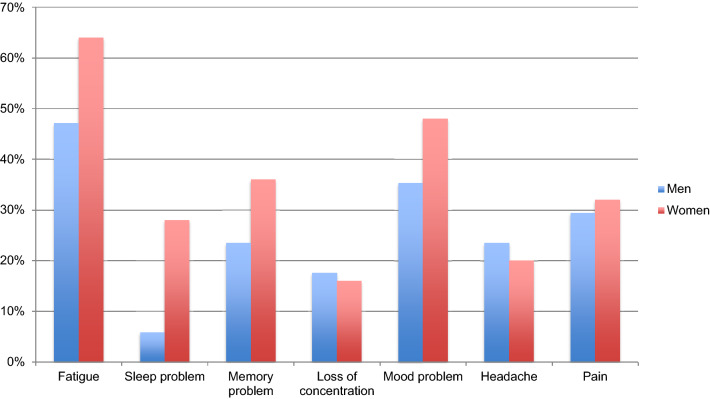
Most frequently spontaneously reported complaints during the interview for both men and women

During the interview, only 2 of the 42 participants (both males) reported not to experience any well-being problems during daily living. Most frequently spontaneously reported complaints are given in Fig. 1.

### Mood

Range, mean (or median) and standard deviation on the POMS and HADS are given in Table 1. One-sample *t*-tests indicated that the mean was significantly different from the mean of the reference groups for all the POMS subscales, with small to large effect sizes (*g* = 0.43—2.29). These results indicate that patients report more depression, anger, fatigue, vigor and tension. One-sample Wilcoxon signed-rank tests indicated that only the median of the depression subscale for women of the HADS was significantly higher than the median of the reference group, with a moderate effect size (*r* = 0.50).

**Table 1:** Range, mean or median and standard deviation scores on the Symptom Checklist-90 (SCL-90), Work and Social Adjustment Scale (WSAS), Profile of Mood States (POMS) and Hospital Anxiety and Depression scale (HADS), and comparisons to reference data

Outcome measure	Test			Hypopituitarism		Reference	*t*	*p*	*g*
				Range	*M* (*SD*)	*M (SD)*			
Well-being	SCL-90	Total	♂	99–218	133.06 (27.83)	114.96 (29.69)	2.68	.02*	0.68
			♀	108—289	157.08 (41.67)	123.10 (34.70)	4.08	< .01**	0.97
		Anxiety	♂	10–22	13.88 (3.41)	12.23 (3.80)	2.00	.06	0.43
			♀	10–29	14.48 (4.24)	13.43 (4.91)	1.24	.23	0.21
		Agoraphobia	♂	7–12	7.82 (1.33)	7.62 (1.66)	0.63	.54	0.12
			♀	7–14	8.52 (2.10)	8.12 (2.79)	0.95	.35	0.14
		Depression	♂	17–36	24.88 (5.37)	20.58 (6.76)	3.30	.01*	0.64
			♀	18–68	31.44 (12.84)	22.89 (8.24)	3.33	< .01**	1.02
		Somatic symptoms	♂	13–40	19.12 (6.95)	15.99 (4.90)	1.86	.08	0.63
			♀	13–40	22.96 (7.10)	17.55 (5.64)	3.81	< .01**	0.95
		Inadequacy of thinking and acting	♂	11–40	17.47 (7.26)	12.48 (4.10)	2.83	.01*	1.20
			♀	11–39	21.92 (6.62)	12.98 (4.45)	6.75	< .01**	1.98
		Interpersonal sensitivity	♂	19–32	24.29 (3.95)	23.66 (7.25)	0.66	.52	0.09
			♀	18–67	28.56 (11.46)	24.83 (8.10)	1.63	.12	0.46
		Hostility	♂	6–10	7.59 (1.28)	7.22 (2.11)	1.19	.25	0.18
			♀	6–21	8.24 (3.22)	7.33 (2.11)	1.41	.17	0.43
		Sleeping problems	♂	3–12	6.24 (2.54)	4.25 (2.04)	3.23	.01*	0.97
			♀	3–15	7.88 (3.72)	4.70 (2.33)	4.27	< .01**	1.34
	WSAS	Total	♂	0–31	10.88 (8.89)	0.72 (2.80)	4.72	< .01**	2.29
			♀	0–37	14.04 (9.7 2)		6.85	< .01**	2.52
		Work	♂	0–8	3.00 (2.48)	0.24 (0.97)	4.60	< .01**	2.05
			♀	0–8	3.56 (2.69)		6.16	< .01**	2.14
		Home management	♂	0–6	1.53 (1.77)	0.13 (0.62)	3.26	.01*	1.52
			♀	0–7	2.64 (2.10)		5.98	< .01**	2.19
		Social leisure	♂	0–6	2.29 (2.09)	0.17 (0.58)	4.20	< .01**	2.11
			♀	0–8	3.40 (2.42)		6.69	< .01**	2.55
		Private leisure	♂	0–5	1.65 (1.77)	0.08 (0.42)	3.66	< .01**	1.92
			♀	0–8	2.32 (2.12)		5.29	< .01**	2.07
		Close relationships	♂	0–6	2.41 (1.94)	0.10 (0.46)	4.92	< .01**	2.58
			♀	0–8	2.12 (2.46)		4.11	< .01**	1.62
Mood	POMS	Depression	♂	8–22	11.82 (3.76)	1.9 (4.4)	10.88	< .01**	2.26
			♀	8–39	15.92 (8.63)	2.6 (4.5)	7.72	< .01**	2.79
		Anger	♂	7–28	12.18 (6.03)	3.7 (4.6)	5.79	< .01**	1.82
			♀	7–31	13.32 (6.40)	3.6 (4.1)	7.60	< .01**	2.29
		Fatigue	♂	6–25	14.06 (5.11)	3.3 (4.4)	8.69	< .01**	2.43
			♀	7–29	16.92 (6.86)	4.6 (5.3)	8.99	< .01**	2.28
		Vigor	♂	11–22	14.29 (3.33)	12.1 (4.5)	2.72	.02*	0.49
			♀	8–23	13.48 (4.30)	11.4 (4.9)	2.42	.02*	0.43
		Tension	♂	6–18	10.88 (3.69)	3.6 (4.0)	8.14	< .01**	1.82
			♀	6–22	11.80 (4.69)	5.1 (4.9)	7.14	< .01**	1.37

			*Hypopituitarism*		*Reference*	*Z*	*p*	*r*
			Range	Median	Median			
HADS	Anxiety	♂	1–11	5	5	0.21	.83	0.05
		♀	1–15	6	6	1.01	.31	0.20
	Depression	♂	0–9	3	3	0.95	.34	0.23
		♀	0–20	4	3	2.51	.01*	0.50

*p ≤ 0.05, **p ≤ 0.01

### Well-being

Range, mean and standard deviation of the scores on the SCL-90 and SAL are given in Table 1. One-sample *t*-tests indicated that both men and women scored significantly worse as compared to the reference mean on SCL-90 total score, depression, inadequacy of thinking and acting and sleeping problems with effect sizes ranging from medium to large (*g* = 0.64 – 1.34). Women also scored higher on somatic symptoms, with a large effect size (*g* = 0.95).

**Fig. 2:**
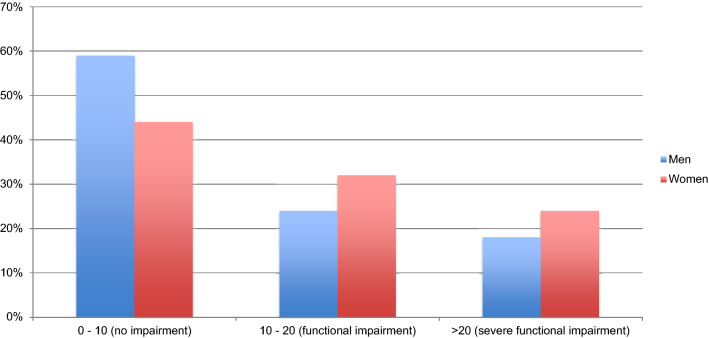
Total score on the Work and Social Adjustment Scale (WSAS) for both men and women summarized in three groups. Data are presented in % of men or women that had scores falling in one of the three total score groups

As seen in Fig. 2, about half of both female and male participants scored between 0 and 10 on the SAL, which is comparable to the score of subclinical patients according to Mundt et al. [60]. The other half of the participants had a score between 11 and 40, which represent significant functional impairment. Within the group of functional impairment, 3 (18%) male and 6 (24%) females had a score that also represented severe limitations, which suggest moderately severe or worse psychopathology. The proportion of patients with testscores corresponding to the no impairment or severe impairment group did not differ by gender, *χ*^2^ (1) = 0.89, *p* = 0.35 respectively *χ*^2^ (1) = 0.24, *p* = 0.62. One sample *t-*tests indicated that the mean was significantly different from the mean of the reference groups for the SAL total score and all subscales for both men and women, with large effect sizes (*g* = 1.52–2.58) as shown in Table 1.

#### Illness perceptions

**Fig. 3:**
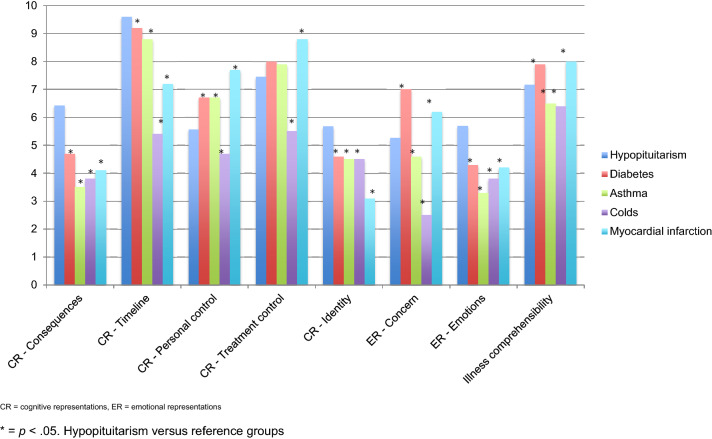
Mean scores and comparisons (one sample *t*-tests) on The Brief Illness Perception Questionnaire (IPQ-B DLV) between hypopituitarism and reference groups

Illness perceptions data of the participants are given in Fig. 3. On the cognitive representations, participants had mainly high scores: timeline (9.6), treatment control (7.5) and consequences (6.4). This is seen as negative illness perceptions for consequences and timeline, but positive for treatment control. Scores on emotional representations, personal control and identity were average. Illness comprehensibility also had an above average score (7.2). Reference data on illness perceptions are also shown for other diseases (diabetes, asthma, colds and myocardial infarction) from a study of Broadbent et al. [63] in Fig. 3. Comparisons on the cognitive and emotional representations were made between hypopituitarism and the reference groups and one sample *t-* tests indicated that, with exception of treatment control in the diabetes and asthma group, all means were significantly different, shown by an asterisk in Fig. 3.

Answers to the open question about illness causalities in the IPQ were categorized to 6 groups: pituitary pathology (e.g. pituitary tumour or hormonal deficiency; 67%), treatment shortcomings (e.g. effect of irradiation, shortcoming of hormonal substitution; 36%), stress (e.g. stress, busy at work; 19%), psychological (e.g. sadness, feeling to lag behind events; 14%), other disease (e.g. diabetes, asthma; 21%) or other (e.g. memory problems, fatigue; 26%).

#### Illness perceptions and psychological well-being

Spearman’s correlation between different aspects of illness perceptions and psychological well-being are showed in Table 2. There were positive, moderate to strong correlations between different aspects of mood and IPQ consequences, identity, concern and emotions. Correlations between different aspects of mood and IPQ treatment control and illness comprehensibility appeared to be negative, and weak to moderate. Also, there were weak to strong correlations between well-being and IPQ consequences, treatment control, identity, concern and emotions.

**Table 2:** Spearman’s correlation (***ρ***) between different aspects of the Illness Perceptions Questionnaire (IPQ) and different aspects of mood (Profile of Mood States; POMS and Hospital Anxiety and Depression; HADS) and well-being (Symptom Checklist-90; SCL-90 and Work and Social Adjustment Scale; WSAS)

	Mood						Well-being	
	POMS—depression	POMS—anger	POMS—fatigue	POMS—tension	HADS—anxiety	HADS -depression	SCL-90 total score	WSAS total score
IPQ—Consequences	.30	.43**	.51**	.30	.21	.47**	.67**	.48**
IPQ- Timeline	.10	− .14	.01	.06	< − .01	− .17	.05	< .01
IPQ—Personal control	− .21	− .10	− .23	.19	− .07	< − .01	− .18	− .25
IPQ—Treatment control	− .19	− .38*	− .36*	− .23	− .40**	− .36*	− .36*	− .27
IPQ—Identity	.12	.30	.50**	.05	.01	.24	.38*	.78**
IPQ—Concern	.29	.41**	.34*	.27	.36*	.18	.27	.34*
IPQ—Illness comprehensibility	− .31*	− .31*	.02	− .39*	− .24	− .28	− .08	− .02

^*^ *p* ≤ 0.05, ** *p* ≤ 0.01

### Causal attributions

**Fig. 4:**
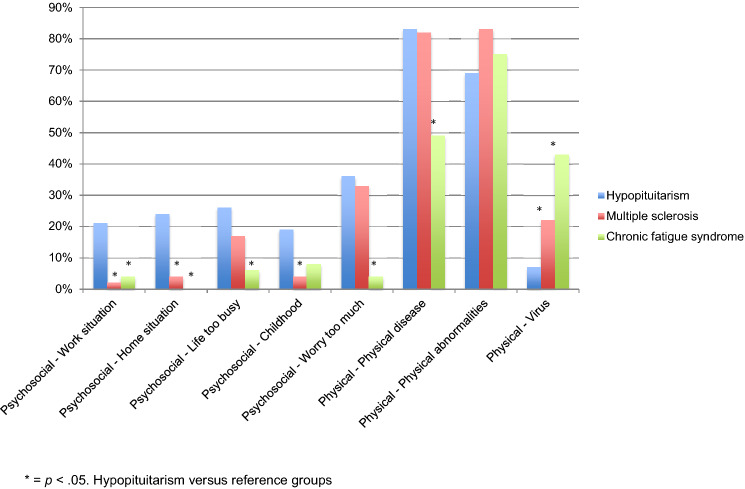
% of participants answering positive on the psychosocial and physical attributions of the Causal Attributions List (CAL) and comparisons (*χ*^2^ test) to reference data: multiple sclerosis and chronic fatigue syndrome

Total scores on physical attributions (*M* = 14.21, *SD* = 3.12) were higher than scores on psychosocial attributions (*M* = 10.07, *SD* = 3.06), meaning participants attributed their complaints more to physical causes than psychological causes. Of all participants, 93% thought that their complaints were due to hormonal disturbances and 79% thought that their complaints persist due to physical cause. When compared to patients with other chronic illnesses (multiple sclerosis and chronic fatigue syndrome; Vercoulen et al. [64]), *χ*^2^ tests indicated that a higher percentage of the present participantgroup thought their complaints were due to psychosocial attributions, but not due to physical attributions, as shown by an asterisk in Fig. 4.

## Discussion

The primary aim of present study was to objectify a spectrum of persisting subjective psychological complaints in adult patients with hypopituitarism, at least six months after normalizing of the hormonal disturbances. It was expected that, when compared to healthy reference groups, these patients show deviating scores on several tests concerning mood and psychological well-being. Secondary, illness perceptions and causal attributions were identified within this patient group. The results of the present study demonstrate that adult patients with hypopituitarism indeed show more negative mood states, score suboptimally on several aspects of psychological well-being and that illness perceptions and well-being are related. This indicates that despite normalization of hormonal disturbances, these patients can still experience problems during daily living.

A great number of participants reported to experience complaints of fatigue and mood problems, which was confirmed by high scores on negative mood states on the POMS subscales depression, anger, fatigue and tension. These reported mood problems are less pronounced on the HADS, in which only depression in women was found. Since the HADS is developed as a screening instrument for clinical mood disorder severity [55], this discrepancy points out that depression in hypopituitarism probably is more of a mood state, rather than an clinical trait. This finding is in line with previous inconclusive outcomes concerning the effect of hypopituitarism on mood and also suggests that, on average, hypopituitarism is related to subtle mood state problems rather than severe mood disorders.

Several aspects of psychological well-being have been found to be affected in the present study. First of all, participants showed a high score on inadequacy of thinking and acting, a dimension in which problems of cognitive functioning are central. This finding is supported by previous results of cognitive dysfunction in treated hypopituitarism [27, 58]. Cognitive dysfunctions were also among most reported problems: 31% reported to have memory complaints and 17% a loss of concentration. As reported during the interview and objectified during the SCL-90, sleeping problems also form a common and considerable complaint within this patient group, which can affect psychological well-being and cognitive functioning. There was no clear association between appropriately suppleted hypopituitarism and sleep complaints yet, which makes this the first study in confirming these relationships. Compared to healthy references, women also had more somatic symptoms such as headache, sore muscles and pain in the lower back. Pain and headache were also frequently mentioned complaints during the interview.

When discussing practical consequences, notable functional impairment due to the hypopituitarism is found in half of the participants. Part of the participants even had a functional impairment score that corresponds with moderate to severe psychopathology. Hence, hypopituitarism has substantial influence on work and daily living for a respectable part of the patient group. Altogether, in patients treated for hypopituitarism, just like other treated pituitary diseases [24], a subnormal score on psychological well-being is found, leading to substantial functional impairment and thereby to a decreased QoL.

Comparisons with gender-related reference groups were made and present study found that, although different aspects of mood and psychological well-being were worse for both genders, women usually were slightly more affected. However, the found difference in outcome measures for gender in this study is less distinct than in previous mentioned studies [27, 52].

Participants generally believed that hypopituitarism had a lot of influence on their lives. Concerning illness control, beliefs were that current treatment had a very mitigating effect on their illness and that participants themselves could control the illness to some extent. They belief to experience physical complaints due to their illness to a mild extent. In general, they were not very concerned about the hypopituitarism and it did affect their state of mind to a mild extent. Besides, participants reported to belief to have good illness comprehensibility. When compared to different reference groups (diabetes, astma, colds and myocardial infarction [64]), these illness perceptions are most alike with a group of diabetic patients. Compared to these other diseases, participants had more negative and realistic illness perceptions on consequence, timeline, identity and emotions. When illness perceptions in hypopituitarism are compared to other pituitary diseases, similarities were found in good understanding of the illness, the belief of a chronic nature of the illness and some lack in personal control over the illness [45, 46]. Compared to a group of patients treated for CD [46] and patients treated for hypopituitarism of the current study, patients treated for acromegaly seem to belief that their illness has less influence on their lives [45]. Besides, patients of the current study tend to belief to have more physical complaints due to their illness than patients treated for acromegaly and CD [45, 46].

Most participants considered pituitary pathology as cause for their illness and additional complaints. More surprisingly, about 25% thought their complaints were due to psychosocial attributions, such as stress and home or work situation. This percentage is higher than for patients with other chronic diseases (multiple sclerosis or chronic fatigue syndrome [64]). Another remarkable result is that more than a third of all participants believed their illness and additional complaints were due to treatment shortcomings, such as effect of irradiation or shortcoming of hormonal substitution. Besides, our results show that there is a relationship between illness perceptions and well-being, with illness perceptions concerning consequences (beliefs about illness effects and outcomes), treatment control (beliefs about treatment effect on illness), concern (about illness), emotions (extent to which the illness affects mood) and illness comprehensibility (extent to which patients believe to understand their disease) being important in particular. By assessing illness perceptions and causal attributions in this patient group, we aim to provide insight for physicians during clinical practice in the extent of the influence of the hypopituitarism in daily living, illness control and feeling of illness comprehensibility. These insights might be helpful when explaining patients about their condition, medical treatment and expected long-term problems. Special attention should be given to clear explanation of the treatment in view of the patient’s knowledge, since more than one third of all participants believed their complaints were due to treatment shortcomings. Eradicating patients’ misunderstandings about hypopituitarism and its treatment and increasing feelings of illness control and comprehensibility can ultimately lead to a greater sense of personal wellbeing. Also, insight in psychological attributions, beliefs about treatment shortcomings and illness perceptions could form a basis for adjustment psychological aspects of treatment.

Advantages of this study include testing a broad spectrum of different aspects of psychological well-being and involve practical consequences, such as influence of the hypopituitarism on work and daily living. One of the limitations of this study is that only 42% of the invited participants decided to participate in the study. Participants differed on the aetiology of hypopituitarism from non-participants; more congenital forms were present in the non-participants. This could form a potential bias since it can be expected that living your entire life with a disease versus developing a disease later in life lead to differences in psychological adjustment. When statements about present results are being made, it should be taken into consideration that current study lacks a control group and longitudinal data. Given that this study lacks a control group, no statements about the direct relationship between psychological well-being and hypopituitarism can be made. Still the design does fit the primary aim of the study, namely expanding knowledge of the sequela of hypopituitarism by identifying psychological well-being and illness perceptions in patients with hypopituitarism. Besides, an advantage of this study is that comparisons are made with large healthy reference groups. Since etiologic groups are represented in small numbers, it is difficult to translate current outcomes of this heterogeneous group to individual patients. Therefore, we performed sensitivity analyses in which we groupwise excluded four important subgroups of patients (cranial radiotherapy, congenital forms of hypopituitarism, Cushing’s disease and acromegaly; data not shown). Excluding these subgroups did not change the results of the current study and we thereby conclude that it is expected that all individual patients can have suboptimal psychological well-being.

Treatment and management of hypopituitarism currently focuses on normalization of hormonal disturbances, rather than functional outcomes such as well-being, mental impairment and QoL [67, 68]. As a consequence, discrepancies may arise between patients expected versus actual well-being. Interestingly, almost all of the objectified outcomes were complaints initially reported by participants during the open interview. This could indicate that, in general, patients with hypopituitarism have a realistic self-insight and are able to assess their well-being in a justified way.

## Conclusions

The results of the present study show that despite normalization of hormonal disturbances, patients with hypopituitarism in general can still experience problems during daily living, including negative mood states and a decreased psychological well-being. Women were slightly more affected. To our knowledge, no other research has been conducted on subjective psychological complaints in patients with treated hypopituitarism. By objectifying these mood problems and other affected aspects of psychological well-being, we hope to have provided a first step towards more understanding of the long-term, persisting mental effects of hypopituitarism which endocrinologists might face at the outpatient clinic. It is recommended to perform a subsequent study, which is directed on the design and evaluation of a psychological-therapeutic intervention. The aim of such an intervention would be the reduction of psychological complaints, such as depression, anger, fatigue and sleeping problems. Besides, the intervention may focus on negative illness perceptions such as consequences, treatment shortcomings and psychosocial attributions (stress, living environment). This intervention may in turn improve QoL and well-being in these patients.

## Data Availability

Research data are not shared.
